# Histone methyltransferase SETD1A interacts with HIF1α to enhance glycolysis and promote cancer progression in gastric cancer

**DOI:** 10.1002/1878-0261.12689

**Published:** 2020-04-26

**Authors:** Jugang Wu, Hongjuan Chai, Xin Xu, Jiwei Yu, Yan Gu

**Affiliations:** ^1^ Department of General Surgery Shanghai Ninth People’s Hospital Shanghai JiaoTong University School of Medicine China; ^2^ Department of Gynecology and Obstetrics Shanghai Ninth People’s Hospital Shanghai JiaoTong University School of Medicine China

**Keywords:** gastric cancer, glycolysis, HIF1α, histone methyltransferase SETD1A, progression

## Abstract

Growing tumors alter their metabolic profiles to support the increased cell proliferation. SETD1A, a histone lysine methyltransferase which specifically methylates H3K4, plays important roles in both normal cell and cancer cell functions. However, the function of SETD1A in gastric cancer (GC) progression and its role in GC metabolic reprogramming are still largely unknown. In the current study, we discovered that the expression of SETD1A was higher in GC tumor specimens compared to surrounding nontumor tissues. Upregulation of SETD1A increased GC cell proliferation, whereas downregulation of SETD1A inhibited GC cell proliferation. Furthermore, knockdown of SETD1A reduced glucose uptake and production of lactate and suppressed glycolysis by decreasing the expression of glycolytic genes, including GLUT1, HK2, PFK2, PKM2, LDHA, and MCT4. Mechanistically, SETD1A interacted with HIF1α to strengthen its transactivation, indicating that SETD1A promotes glycolysis through coactivation of HIF1α. SETD1A and HIF1α were recruited to the promoter of HK2 and PFK2, where SETD1A could methylate H3K4. However, knockdown of SETD1A decreased the methylation of H3K4 on HK2 and PFK2 promoter and reduced HIF1α recruitment necessary to promote transcription of glycolytic genes. Inhibition of HIF1α decelerated SETD1A‐enhanced GC cell growth. In additional, there was a linear correlation between SETD1A and several key glycolytic genes in human GC specimens obtained from TCGA dataset. Thus, our results demonstrated that SETD1A interacted with HIF1α to promote glycolysis and accelerate GC progression, implicating that SETD1A may be a potential molecular target for GC treatment.

## Graphical abstract

SETD1A is upregulated in gastric cancer tissues. Knockdown of SETD1A decelerates gastric cancer glycolysis. SETD1A increases the methylation of H3K4 on HK2 and PFK2 promoter and enhances HIF1α recruitment necessary to promote transcription of glycolytic genes. Inhibition of SETD1A reduces gastric cancer development.

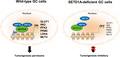

**Abbreviations**
CCK‐8: cell counting kit‐8
ChIP: chromatin immunoprecipitation
co‐IP: co‐immunoprecipitation
GC: gastric cancer
GLUT1: glucose transporter type 1
HIF1α: hypoxia‐inducible factor 1 subunit alpha
HK2: hexokinase 2
HRE: hypoxia response element
KMT: lysine methyltransferase
LDHA: lactate dehydrogenase A
MCT4: monocarboxylate transporter 4
PFK2: 6‐phosphofructo‐2‐kinase
PKM2: pyruvate kinase M2
RT‐qPCR: real‐time quantitative polymerase chain reaction
TCGA: The Cancer Genome Atlas
TNM: tumor node metastasis

## 1 Introduction

Gastric cancer (GC) is the third leading cause of cancer‐associated death among the world (Bray *et al.*, 2018) and the second leading cause of cancer‐associated death in China (Chen *et al.*, 2016). Though the main treatments for GC include surgery, targeted therapy, chemotherapy, and radiotherapy, the prognosis of GC remains dismal (Mao *et al.*, 2019). Approximately a half of GC patients will suffer tumor recurrence after radical resection (Huang *et al.*, 2009; Spolverato *et al.*, 2014; Wu *et al.*, 2003). The main factors of death are local recurrences and distant metastases in GC patients (Deng *et al.*, 2011). Despite marked advances in nearest years, our understanding of GC progression remains largely restricted.

Unlike normal cells, tumor cells mainly rely on glycolysis to produce energy needed for cellular processes even in the presence of oxygen conditions. This process referred to aerobic glycolysis or the Warburg effects, which avidly take up glucose, broken glucose down to form pyruvate, and converted pyruvate to lactate, is considered as a hallmark of tumor cells (Koppenol *et al.*, 2011; Payen *et al.*, 2016). Increasing evidences showed that a key transcription factor hypoxia‐inducible factor 1α (HIF1α), which was induced by oncogenes including Ras and Src, upregulated the glucose transporters GLUT1 and GLUT3, most of the glycolytic enzymes, and lactate dehydrogenase A (LDHA) that catalyzes the conversion of pyruvate to lactate, resulting in metabolic reprogramming (Dang and Semenza, 1999; Fantin *et al.*, 2006). Aberrant activation of HIF1α and related aerobic glycolysis promoted different steps of the cancer development, including GC (Payen *et al.*, 2016). Therefore, specifically inhibition of HIF1α‐associated metabolic pathway has been presumed as a valuable therapeutic strategy for GC progression.

Chromatin modification was emerging as key mediators of tumorigenesis. Histone methylation, catalyzed by lysine methyltransferases (KMT) and inhibited by lysine demethylase, has been linked to both transcriptional activation (H3K4, H3K36, and H3K79) and repression (H3K9, H3K27, and H4K20; Black *et al.*, 2012; Tajima *et al.*, 2015). Previous reporters have shown that abnormal methylation of H3K4 was occurred in various tumors (Chervona and Costa, 2012; Ellinger *et al.*, 2010; Khan *et al.*, 2015; Seligson *et al.*, 2009). SETD1A, a H3K4 methyltransferase, was upregulated and promoted cancer progression including colorectal cancer and breast cancer (Salz *et al.*, 2015; Salz *et al.*, 2014; Shilatifard, 2012; Tajima *et al.*, 2015). However, the function of SETD1A in GC progression was still largely unknown. The object of the current study was to identify the function and underlying mechanism of SETD1A in GC progression.

In this report, we found that SETD1A was upregulated in GC patients and contributed to GC cell proliferation and tumorigenesis. In addition, we also showed that SETD1A upregulated the expression of HIF1α target genes through interacting with HIF1α to enhance glycolysis in GC cells. In conclusion, we indicated SETD1A as a major regulator of HIF1α‐mediated glycolysis and GC progression.

## 2 Methods

### 2.1 Patients and samples

Eighteen paired human GC tissue samples were taken from the tumor area and adjacent nontumorous stomach of GC patients who underwent stomach resection for sporadic GC (all with TNM stages II–III) at the Shanghai Ninth People’s Hospital, Shanghai JiaoTong University School of Medicine. All the patients had never received radiotherapy or chemotherapy before undergoing stomach resection. The human studies were approved by the Translational Medical Independent Ethics Committee of Shanghai Ninth People’s Hospital (Shanghai, China). The experiments were undertaken with the understanding and written consent of each subject. The study methodologies conformed to the standards set by the Declaration of Helsinki.

### 2.2 Bioinformatic analysis of clinical data

Gastric cancer dataset was obtained from The Cancer Genome Atlas (TCGA). The expression of SETD1A and correlation were assessed by Gene Expression Profiling Interactive Analysis (GEPIA) (http://gepia.cancer-pku.cn/; Tang *et al.*, 2017). Overall survival of GC patients was assessed by KM plotter: Kaplan–Meier plotter (http://kmplot.com/analysis/) (Nagy *et al.*, 2018). The GEO dataset included http://www.ncbi.nlm.nih.gov/geo/query/acc.cgi?acc=GSE24120, http://www.ncbi.nlm.nih.gov/geo/query/acc.cgi?acc=GSE15459, http://www.ncbi.nlm.nih.gov/geo/query/acc.cgi?acc=GSE22377, http://www.ncbi.nlm.nih.gov/geo/query/acc.cgi?acc=GSE29272, http://www.ncbi.nlm.nih.gov/geo/query/acc.cgi?acc=GSE51105, and http://www.ncbi.nlm.nih.gov/geo/query/acc.cgi?acc=GSE62254.

### 2.3 Cell lines

The human GC cell lines BGC‐823 and AGS, and HEK293T were purchased from the Cell Bank of Type Culture Collection of Chinese Academy of Sciences (Shanghai, China) and were maintained in RPIM 1640 medium or DMEM replenishing 10% FBS (Gibco, Cat# 10099141) and 1% penicillin–streptomycin.

### 2.4 SETD1A‐knockdown experiments

Two special small interfering RNAs (siRNAs) against human SETD1A were synthesized. Each targeting sequences were shown as follows: siSETD1A‐1, 5′‐GGAAAGAGCCATCGGAAATTT‐3′; siSETD1A‐2, 5′‐GACAACAACGAATGAAATATT‐3′. The targeting sequences were inserted into pll3.7‐puro construct to generate pll3.7‐puro‐shSETD1A. All constructs were transfected into BGC‐823 and AGS cells by Lipofectamine 2000 (Invitrogen, Carlsbad, CA, USA, Cat#11668019) according to the manufacturer’s instructions.

### 2.5 Western blot

Cells were lysed by RIPA buffer, and 20 μg of total protein was separated and transferred onto NC membranes. The membranes were probed with the following primary antibodies: anti‐SETD1A (Bethyl, Montgomery, TX, USA, Cat#A300‐289A), anti‐HIF1α (BD, Franklin lake, NJ, USA, Cat#610959), anti‐HK2 (Santa Cruz, Santa Cruz, CA, USA, Cat#sc‐130358), anti‐LDHA (Santa Cruz, Cat#sc‐133123), anti‐PCNA (CST, Danvers, MA, USA, Cat#2586s), Flag (Sigma, Burlington, MA, USA, Cat#F1804), HA (Sigma, Cat#H9658), and anti‐β‐actin (Sigma, Cat#A5316) at 4 °C overnight. Membranes were then probed with appropriated horseradish peroxidase (HRP)‐conjugated secondary antibody and visualized by chemiluminescence.

### 2.6 Cell counting Kit‐8 (CCK‐8) assay

A total of 2,000 BGC‐823 and AGS cells were seeded in 96‐well plates, and cell growth was detected every day by Cell Counting Kit‐8 (CCK‐8) (Dojindo, Kumamoto, Japan, CK04) assay on a microplate spectrophotometer reading system at a wavelength setting of 450 nm.

### 2.7 Glucose uptake, lactate, and pH value measurements

The procedures for measuring glucose uptake, lactate, and pH values in culture medium of BGC‐823 and AGS cells were described previously (Wan *et al.*, 2017). Briefly, SETD1A‐knockdown and control BGC‐823 cells were treated with normoxic (21% O_2_) or hypoxic (1% O_2_) conditions for 24 h. The medium was collected and removed the cells by centrifugation. Glucose uptake, lactate levels, and pH values in the media were measured by the Glucose Uptake Colorimetric Assay Kit (BioVision, Milpitas, CA, USA, Cat#K676), the Lactate Assay Kit (BioVision, Cat#627), and pH meter following the manufacturer’s instructions, respectively.

### 2.8 Real‐time quantitative PCR (RT‐qPCR)

Total RNAs were isolated using TRIzol (Invitrogen), and 2 μg of total RNA was reversed to cDNA by gDNA Erase and PrimeScript RT Reagent Kits (TAKARA Biotechnology, Dalian, China) following the manufacturer’s instructions, respectively. SYBR Green‐based RT‐qPCR was done using a Bio‐Rad PCR instrument. The RT‐qPCR primer sequences are shown below (Zhao *et al.*, 2014): SETD1A: forward, 5′‐TTGCCATGTCAGGTCCAAAAA‐3′, reverse, 5′‐CGTACTTACGGCACATATCCTTC‐3′; GLUT1: forward, 5′‐GATTGGCTCCTTCTCTGTGG‐3′, reverse, 5′‐TCAAAGGACTTGCCCAGTTT‐3′; HK2: forward, 5′‐GAGCCACCACTCACCCTACT‐3′, reverse, 5′‐CCAGGCATTCGGCAATGTG‐3′; PFK2: forward, 5′‐ATTGCGGTTTTCGATGCCAC‐3′, reverse, 5′‐GCCACAACTGTAGGGTCGT‐3′; PKM2: forward, 5′‐ATGTCGAAGCCCCATAGTGAA‐3′, reverse, 5′‐TGGGTGGTGAATCAATGTCCA‐3′; LDHA: forward, 5′‐AAGCGGTTGCAATCTGGATTCAG‐3′, reverse, 5′‐GGTGAACTCCCAGCCTTTCC‐3′; MCT4: forward, 5′‐CAGTTCGAGGTGCTCATGG‐3′, reverse, 5‐ATGTAGACGTGGGTCGCATC‐3; and β‐actin: forward, 5′‐AGCGAGCATCCCCCAAAGTT‐3′, reverse, 5′‐GGGCACGAAGGCTCATCATT‐3′.

### 2.9 Luciferase assay

HEK293T or BGC‐823 cells were transfected with HK2 promoter–reporter plasmid which has HRE using Lipofectamine 2000. Renilla luciferase plasmid was acted as a transfection efficiency control (Zhao *et al.*, 2014). After 48 h, cells were harvested and luciferase activity was determined using the Dual‐Luciferase Reporter Assay System (Promega, Madison, WI, USA, Cat#1910).

### 2.10 Co‐immunoprecipitation assay

Cells were firstly lysed with IP lysis buffer (Wan *et al.*, 2017). Cell lysates were immunoprecipitated using the FLAG (Sigma, Cat#F1804), HA (Sigma, Cat#H9658), anti‐SETD1A (Bethyl, Cat#A300‐289A), anti‐HIF1α (BD, Cat#610959) antibodies, or control immunoglobulin G (IgG). After extensive washing, precipitates were performed by western blot analysis.

### 2.11 Chromatin immunoprecipitation (ChIP) assay

Control and SETD1A‐knockdown BGC‐823 cells were cultured under normoxic or hypoxic condition for 24 h, and then, the chromatin was immunoprecipitated by anti‐SETD1A (Bethyl, Cat#A300‐289A), anti‐HIF1α (BD, Cat#610959), anti‐H3K4me3 (Abcam, Cat#ab8580) antibodies, or nonspecific IgG (Santa Cruz). ChIP DNA was purified by SimpleChIP^®^ Enzymatic Chromatin IP Kit (CST, Cat#9003) according to the manufacturer’s instructions and amplified by real‐time PCR for the HK2 and PFK2 promoter. Primers were as follows: HK2: forward, 5′‐AGGAGTAAGACAAGGGCAGG‐3′, reverse, 5′‐CACTTCAGCGTCCCAAATAG‐3′; PFK2: forward, 5′‐AGTCTGATACAGGCGGGATG‐3′, reverse, 5′‐AGTGTCAGCGAAGCAGGAAG‐3′. The genomic locations for primers of HK2 promoter are at 74832167 to 74832375 on the chromosome 2. The genomic locations for primers of PFK2 promoter are at 6142945 to 6143157 on the chromosome 10.

### 2.12 Xenograft tumor

Four‐ to six‐week‐old male nude mice were purchased from SLAC Laboratory Animal Co., Ltd. (Shanghai, China). A total of 5 × 10^6^ control or SETD1A‐knockdown BGC‐823 cells were subcutaneously injected into nude mice. The number of mice in each group is six. The tumor size was monitored every 2 days using a vernier caliper from day 5 after injection (Wan *et al.*, 2017). The tumor volume was calculated using the following formula: volume = 0.52 × length × width^2^. All animal experiments were according to the ethic regulations and approved by the Translational Medical Independent Ethics Committee of Shanghai Ninth People’s Hospital (Shanghai, China).

### 2.13 Statistical analysis

All the results were obtained from three or more independent experiments per experiment. The data were expressed as mean ± SD. Statistically significant differences (*P* < 0.05) were examined using *t*‐test.

## 3 Results

### 3.1 SETD1A is overexpressed in GC specimens and predicts poor prognosis

**Fig. 1:**
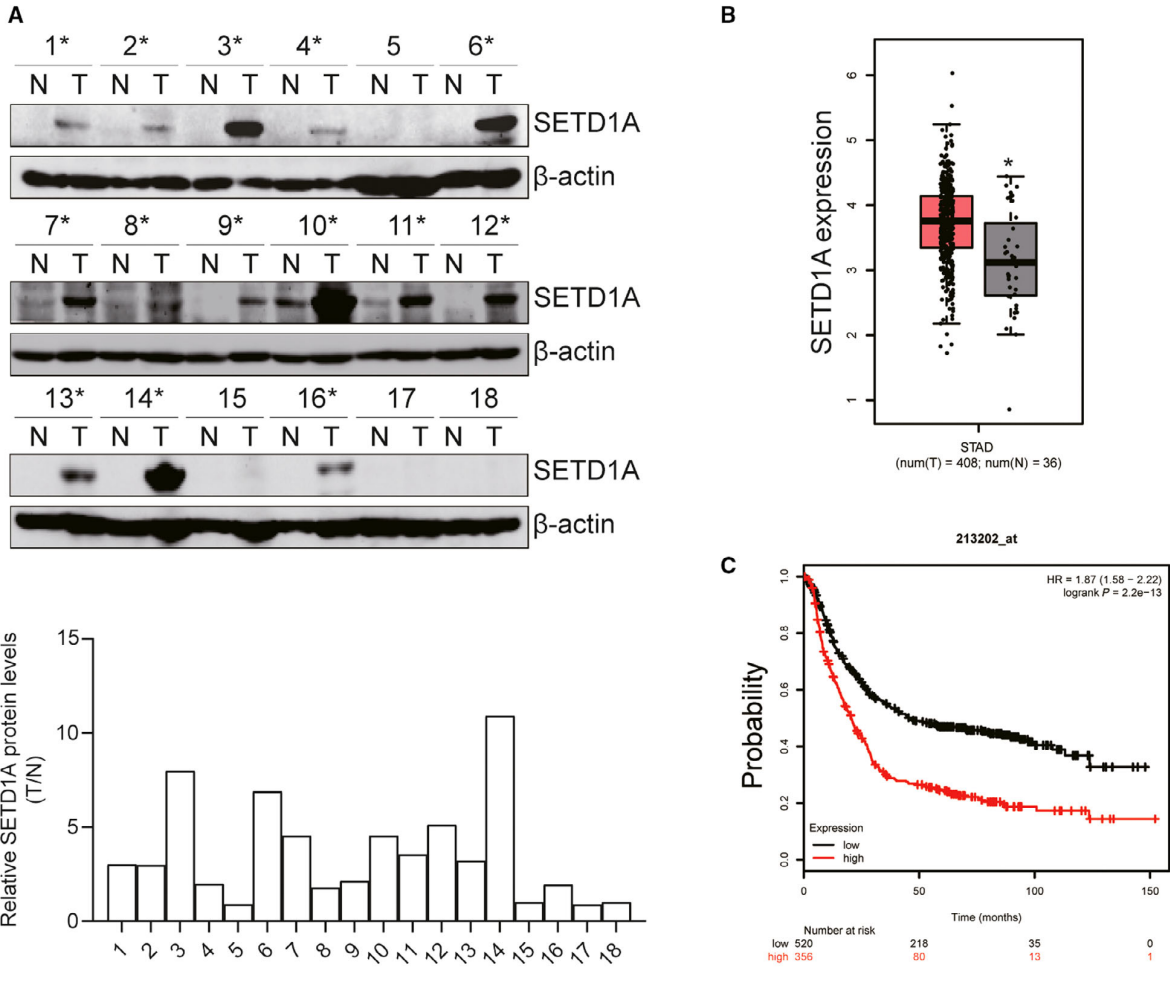
SETD1A was overexpressed in human GC specimens and predicated poor outcome. (A) Analysis of the protein of SETD1A in 18 pairs of GC specimens and surrounding nontumor tissues. (B) Analysis of the expression of SETD1A in normal (*n* = 36) and GC (*n* = 408) specimens from TCGA dataset obtained by GEPIA. (C) Analysis of the overall survival rate in SETD1A‐high and SETD1A‐low expression GC patients from GEO dataset. *P < 0.05.

To determine the role of SETD1A in human GC progression, we detected the protein expression of SETD1A in human GC specimens and the surrounding nontumorous stomach tissues. The western blot results showed that the expression of SETD1A was markedly higher in human GC specimens compared with nontumorous tissues (Fig. 1A). Next, we further analyzed SETD1A expression in normal human stomach specimens (*n* = 36) and GC specimens (*n* = 408) from TCGA dataset. Compared to normal tissues, the expression of SETD1A was significantly upregulated in GC specimens (Fig. 1B). Furthermore, the overall survival rate in SETD1A‐high patients was much lower than in SETD1A‐low patients (Fig. 1C). These results indicated that SETD1A may promote GC progression.

### 3.2 SETD1A promotes GC cell proliferation

**Fig. 2:**
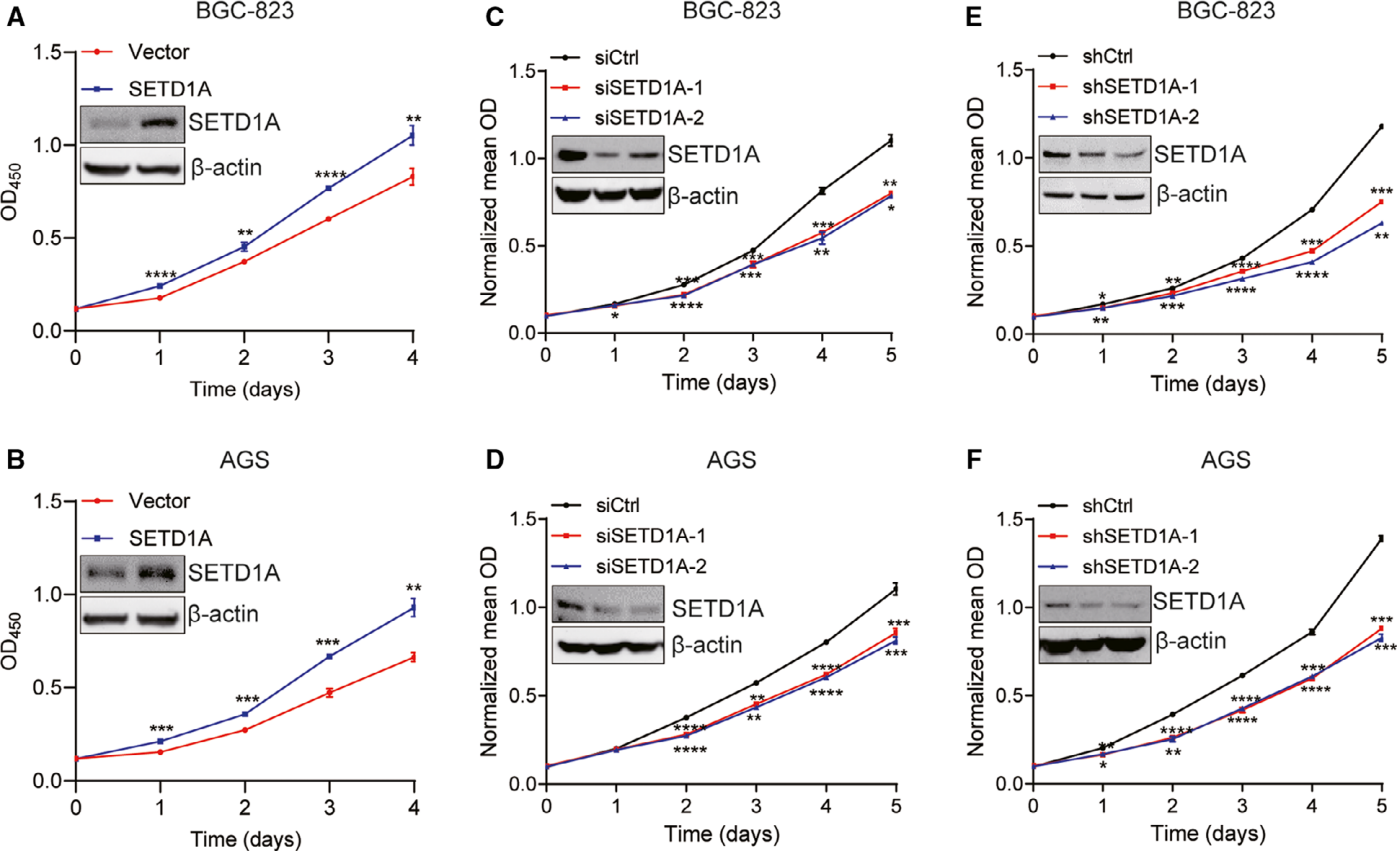
SETD1A promotes gastric cancer cell proliferation. (A, B) Overexpression of SETD1A increased BGC‐823 and AGS cell proliferation. BGC‐823 and AGS cells were transfected with SETD1A plasmid for 48 h and then seeded into 96‐well plates for CCK‐8 assays (mean ± SEM; *n* = 4; Student’s *t*‐test). (C, D) Knockdown of SETD1A decreased BGC‐823 and AGS cell proliferation. Cells were transfected with SETD1A siRNAs for 48 h and then seeded into 96‐well plates for CCK‐8 assays (mean ± SEM; *n* = 4; Student’s *t*‐test). (E, F) Stable SETD1A‐knockdown BGC‐823 and AGS cells grown slowly compared to control cells (mean ± SEM; *n* = 4; Student’s *t*‐test). (mean ± SEM; *n* = 4; Student’s *t*‐test) *P < 0.05, **P < 0.01, ***P < 0.001, ****P < 0.0001.

To detect the function of SETD1A in GC cell proliferation, SETD1A expression plasmids or vector plasmids were transfected into two human GC cell lines, BGC‐823 and AGS cells, respectively. As shown in Fig. 2A,B, the ectopic expression of SETD1A increased proliferation rate of BGC‐823 and AGS cells. This evidence suggested that upregulation of SETD1A could promote GC cell proliferation.

Next, two different special siRNAs against human SETD1A were used to downregulate SETD1A expression in BGC‐823 and AGS cells, respectively. The reduction of SETD1A markedly slows down the proliferation rate in both BGC‐823 and AGS cells (Fig. 2C,D). This suggested that SETD1A is required for GC cell proliferation.

Furthermore, stable SETD1A‐knockdown cell lines were established for further study, and BGC‐823 and AGS cells were transfected with two different special human SETD1A‐knockdown shRNA (pll3.7‐sh‐SETD1A‐1 and pll3.7‐sh‐SETD1A‐2), respectively. The SETD1A‐knockdown stable clones were selected by puromycin. As shown in Fig. 2E,F, two stable SETD1A‐knockdown cell lines were constructed. Same with the results obtained from transient transfection, stable knockdown of SETD1A also markedly reduced the proliferation of BGC‐823 (Fig. 2E) and AGS (Fig. 2F) cells. These data indicated that SETD1A plays a promoting role in GC cell proliferation.

### 3.3 Knockdown of SETD1A suppresses glycolysis and lactate fermentation

**Fig. 3:**
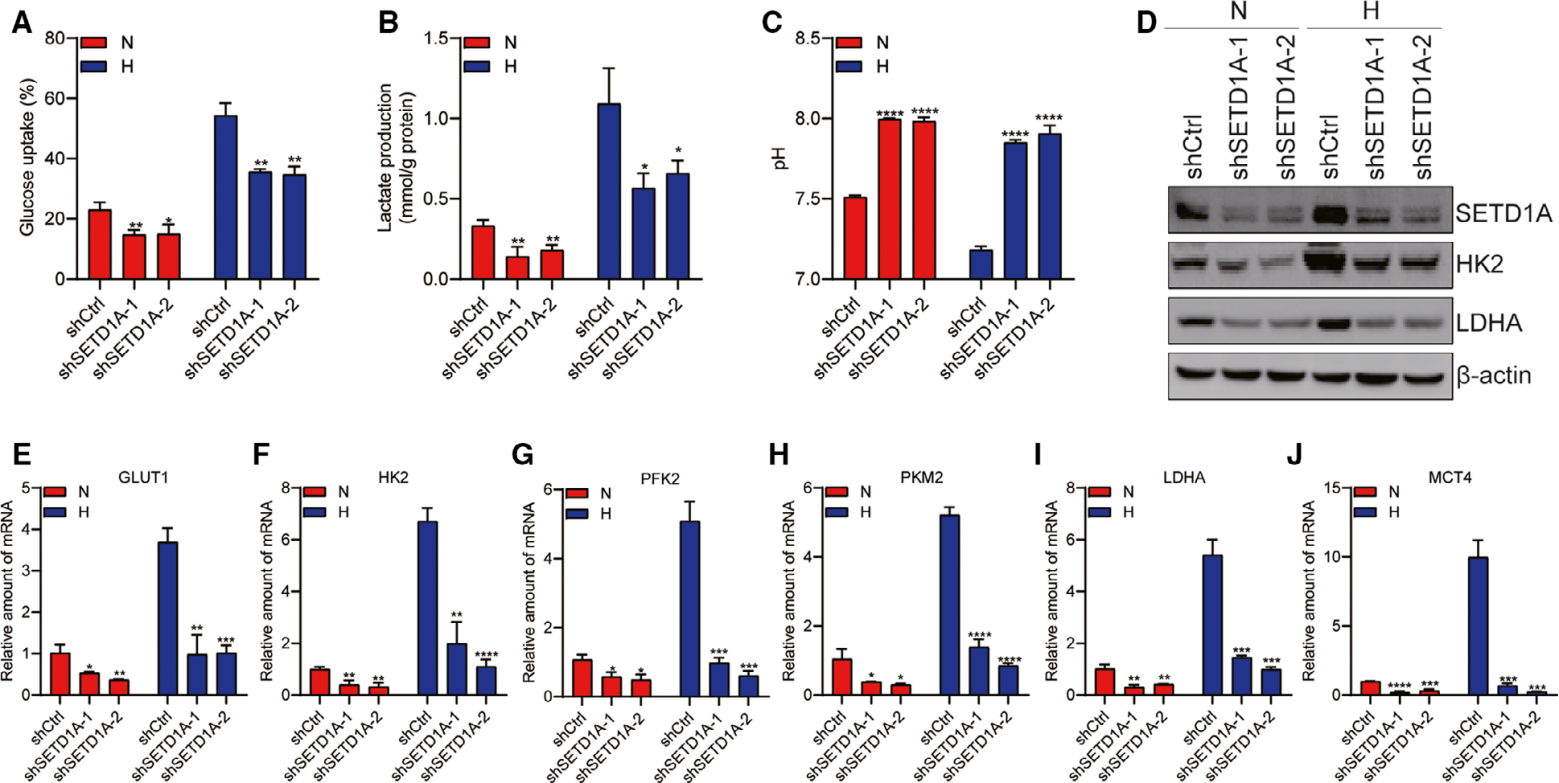
Downregulation of SETD1A decreases glycolysis in BGC‐823 cells. (A–C) Glucose uptake (A), lactate production (B), and pH values (C) of SETD1A‐knockdown cells were reduced under normoxic (N) and hypoxic (H) conditions for 24 h compared to control BGC‐823 cells (mean ± SEM; *n* = 3; Student’s *t*‐test). (D) The protein levels of HK2 and LDHA were remarkedly reduced in SETD1A‐knockdown BGC‐823 cells under normoxic (N) and hypoxic (H) conditions for 24 h. (E–J) The mRNA levels of GLUT1 (E), HK2 (F), PFK2 (G), PKM2 (H), LDHA (I), and MCT4 (J) were remarkedly reduced in SETD1A‐knockdown BGC‐823 cells under normoxic (N) and hypoxic (H) conditions for 24 h (mean ± SEM; *n* = 4; Student’s *t*‐test). *P < 0.05, **P < 0.01, ***P < 0.001, ****P < 0.0001.

Cancer cells use high aerobic glycolysis to satisfy the requirement for proliferation. To reveal the mechanism by which SETD1A promoted GC cell proliferation, the glucose metabolism was monitored to assess whether knockdown of SETD1A decelerated glycolysis. Control and SETD1A‐knockdown BGC‐823 cells were treated with normoxia or hypoxia for 24 h, respectively, and then measure the glucose uptake, lactate, and pH values in the medium. As shown in Fig. 3A–C and Supplementary Fig. 1A–C, knockdown of SETD1A in BGC‐823 (Fig. 3A–C) and AGS (Fig. S1A–C) cells reduced glucose uptake and lactate production and led to rise the pH value under both normoxic and hypoxic conditions, suggesting that SETD1A knockdown reduced glucose metabolism.

To understand the potential mechanism of SETD1A‐mediated glucose metabolism, some key glycolytic gene expression in SETD1A‐knockdown and control BGC‐823 and AGS cells was examined. Knockdown of SETD1A reduced HK2 and LDHA protein levels (Fig. 3D and Fig. S1D) and GLUT1, HK2, PFK2, PKM2, LDHA, and MCT4 mRNA levels under both normoxic and hypoxic conditions in BGC‐823 (Fig. 3E–J) and AGS (Fig. S1E–J) cells. These results indicate that knockdown of SETD1A reduced glucose metabolism by downregulating the expression of glycolytic genes.

### 3.4 SETD1A strengthens glycolysis through coactivation of HIF1α

**Fig. 4:**
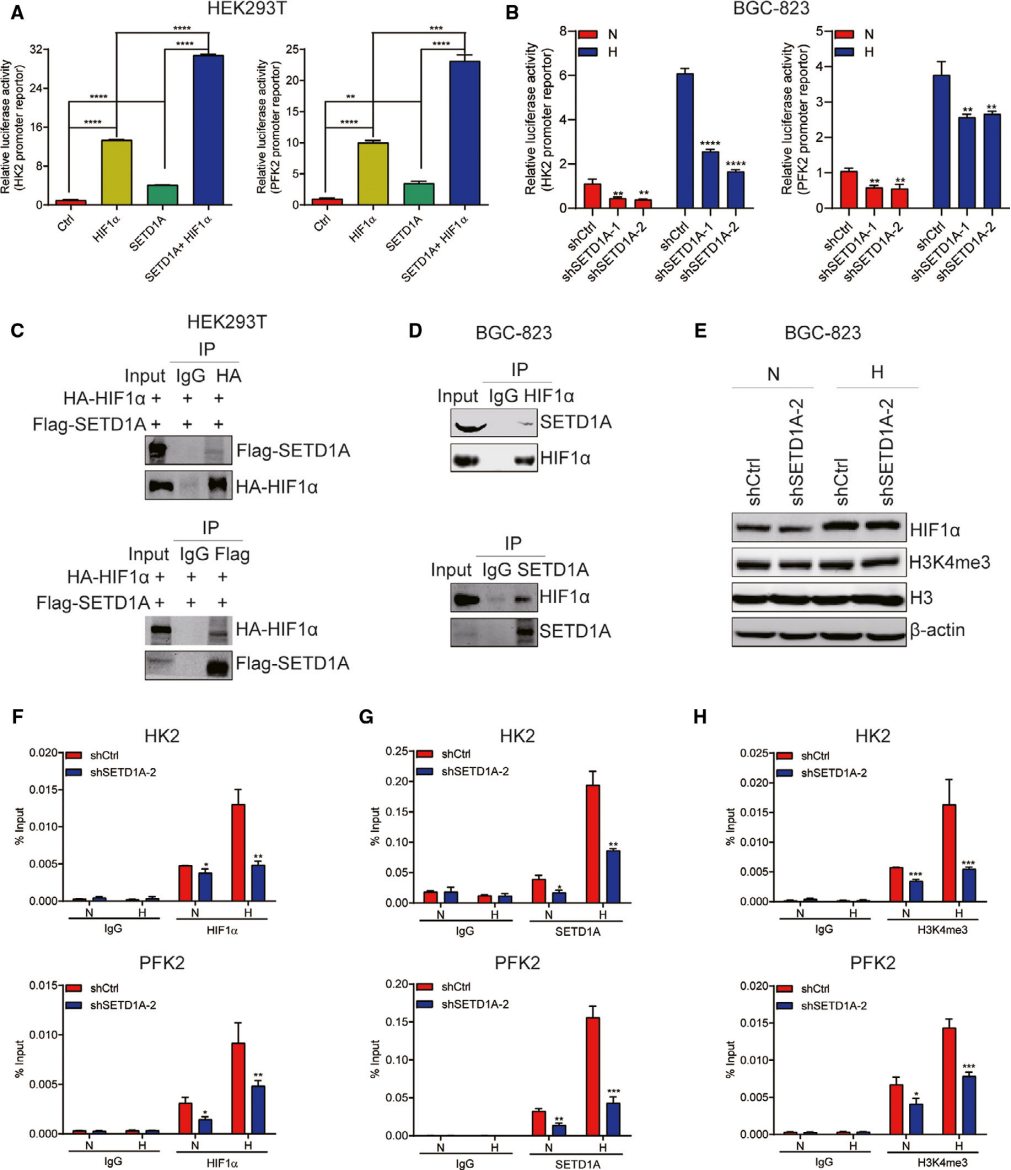
SETD1A cooperates with HIF1α to enhance HIF1α transactivation. (A) SETD1A cooperated with HIF1α to enhance the HK2 and PFK2 promoter–reporter activity (mean ± SEM; *n* = 3; Student’s *t*‐test). (B) Downregulation of SETD1A decreased the HK2 and PFK2 promoter activity in BGC‐823 cells. SETD1A‐knockdown and control BGC‐823 cells were transfected with HRE‐Luc under normoxic (N) and hypoxic (H) conditions. Luciferase activity was measured 48 h after transfection (mean ± SEM; *n* = 3; Student’s *t*‐test). (C) Co‐IP analysis of the interaction between Flag‐SETD1A and HA‐HIF1α. (D) Co‐IP analysis of the interaction between endogenous SETD1A and HIF1α in BGC‐823 cells. (E) Downregulation of SETD1A did not change the expression of HIF1α and global H3K4me3 protein in BGC‐823 cells. (F, G) Downregulation of SETD1A reduced the levels of HIF1α (F), SETD1A (G), and H3K4me3 (H) on the HK2 and PFK2 promoter in BGC‐823 cells. ChIP analysis of the levels of HIF1α (F), SETD1A (G), and H3K4me3 (H) on the HK2 and PFK2 promoter in SETD1A‐knockdown and control BGC‐823 cells under normoxic (N) or hypoxic (H) condition (mean ± SEM; *n* = 3; Student’s *t*‐test). *P < 0.05, **P < 0.01, ***P < 0.001, ****P < 0.0001.

It has been reported that HIFα enhanced glycolysis through promoting the transcription of key glycolytic genes (Chen *et al.*, 2019; Jia *et al.*, 2019; Zhou *et al.*, 2018). We hypothesized that whether SETD1A cooperated with HIF1α to enhance glycolysis. To test this hypothesis, human SETD1A and/or HIF1α expression plasmids, together with the HK2 or PFK2 promoter–reporter which has HRE elements of HIF1α, were transfected into HEK293T cells; then, luciferase activity assay was performed. As shown in Fig. 4A, overexpression of HIF1α or SETD1A alone raised the reporter activity of HK2 and PFK2 promoters, respectively, while co‐overexpression of SETD1A and HIF1α together enhanced HIF1α‐induced reporter activation, indicating that SETD1A could cooperate with HIF1α to induce HIF1α transactivation. This notion was further demonstrated by the findings that the HK2 and PFK2 promoter–reporter activities were lower in SETD1A‐knockdown BGC‐823 cells than control cells with or without hypoxic condition (Fig. 4B).

To detect whether SETD1A can interact with HIF1α, FLAG‐SETD1A and HA‐HIF1α expression plasmids were cotransfected into HEK293T cells and then performed co‐immunoprecipitation (co‐IP) assays by using anti‐HA and anti‐FLAG tag antibodies, respectively. As shown in Fig. 4C, the anti‐FLAG antibody could precipitate HA‐HIF1α (Fig. 4C). Reciprocally, the anti‐HA antibody could also precipitate FLAG‐SETD1A (Fig. 4C). These data indicate that exogenous expressed SETD1A can interact with HIF1α. Furthermore, the endogenous HIF1α and SETD1A interaction could be also detected in BGC‐823 cells (Fig. 4D). These results indicated that SETD1A cooperated with HIF1α to enhance glycolysis.

### 3.5 SETD1A increases H3K4me3 levels at the hypoxia response element on the HK2 and PFK2 promoter to promote HIF1α transactivation

Since SETD1A can increase H3K4me3 levels, we detected whether SETD1A knockdown could change the global H3K4me3 protein levels. The global H3K4me3 protein levels were measured under normoxic and hypoxic conditions. As expected, hypoxia could increase the HIF1α expression (Fig. 4E); however, knockdown of SETD1A did not change the expression of HIF1α and global H3K4me3 protein (Fig. 4E) in BGC‐823 cells. Previous data have indicated SETD1A could assist HIF1α to enhance the HRE reporter activity; we next examined whether HIF1α and SETD1A could recruit to the hypoxia response element (HRE) on the promoter of HIF1α‐regulated glycolytic genes HK2 and PFK2. Knockdown of SETD1A and control BGC‐823 cells was treated with normoxia or hypoxia for 24 h, respectively, and then performed chromatin immunoprecipitation (ChIP) assays to detect the recruitments. The results showed that hypoxia increased HIF1α (Fig. 4F) and SETD1A (Fig. 4G) binding to the HK2 and PFK2 promoter and then methylated the H3K4 on the HK2 and PFK2 promoter (Fig. 4H), which facilitates HIF1α‐mediated HK2 and PFK2 transcription. Whereas SETD1A recruitment on the HK2 and PFK2 promoter was reduced in SETD1A‐knockdown cells as expected (Fig. 4G), SETD1A knockdown also significantly reduced the HIF1α recruitment (Fig. 4F) and H3K4me3 levels (Fig. 4H) on the HK2 and PFK2 promoter. These data indicated that SETD1A could increase H3K4me3 levels on the HK2 and PFK2 promoter to facilitate HIF1α transactivation.

### 3.6 Inhibition of HIF1α‐related glycolytic pathway inhibits SETD1A‐mediated GC cell proliferation

**Fig. 5:**
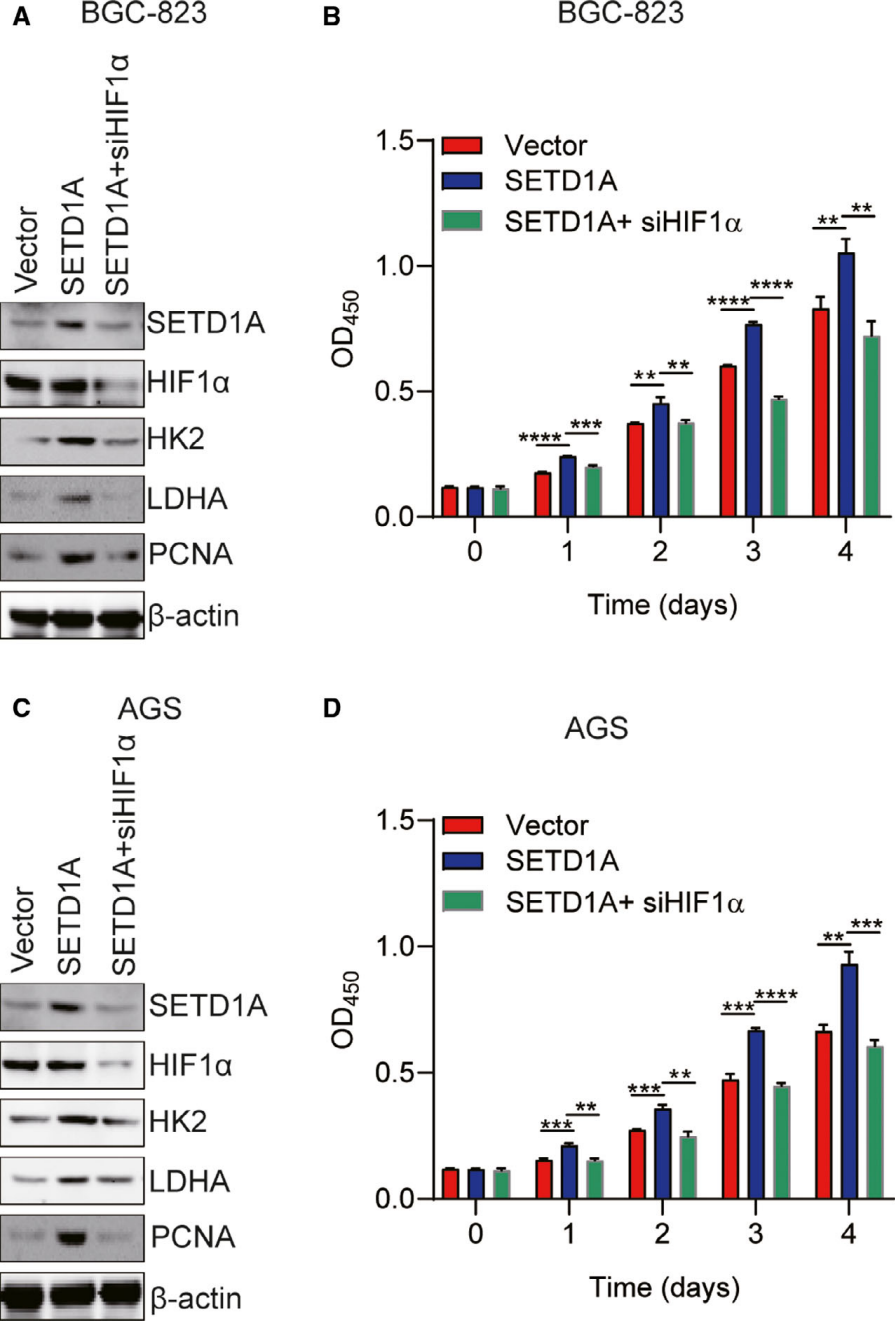
Inhibition of HIF1α suppresses SETD1A‐enhanced GC cell proliferation. (A, B) Knockdown of HIF1α suppressed glycolytic pathway and SETD1A‐enhanced BGC‐823 cell proliferation (mean ± SEM; *n* = 3; Student’s *t*‐test). (C, D) Knockdown of HIF1α suppressed glycolytic pathway and SETD1A‐enhanced AGS cell proliferation (mean ± SEM; *n* = 4; Student’s *t*‐test). **P < 0.01, ***P < 0.001, ****P < 0.0001.

To confirm the key role of HIF1α‐related glycolytic pathway in SETD1A‐induced GC cell proliferation, HIF1α special siRNA was used to knock down the expression of HIF1α in SETD1A‐overexpressing BGC‐823 and AGS cells, and then, CCK‐8 assay was performed to detect the GC cell proliferation. The results showed that knockdown of HIF1α led to suppressing SETD1A‐enhanced HK2, LDHA and PCNA expression, and cell proliferation in SETD1A‐overexpressing BGC‐823 (Fig. 5A,B) and AGS cells (Fig. 5C,D). These results suggested that SETD1A plays a promotional effect on GC cell proliferation by strengthening HIF1α‐related glycolytic pathway.

### 3.7 Knockdown of SETD1A in BGC‐823 cells inhibits tumorigenesis in vivo

**Fig. 6:**
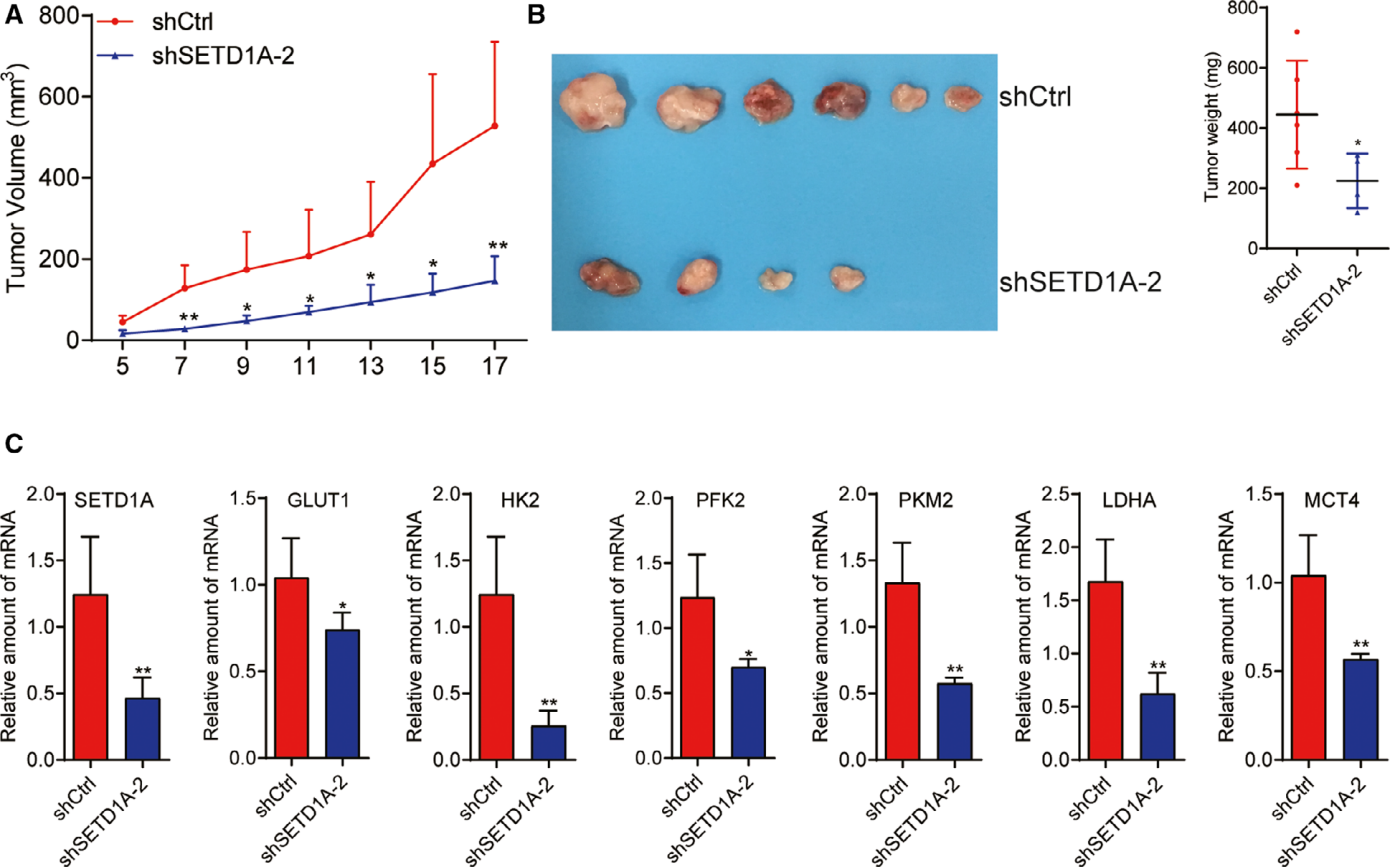
Knockdown of SETD1A reduces gastric cancer cell tumorigenesis. (A, B) Downregulation of SETD1A in BGC‐823 cells inhibited xenograft tumor growth (A) in nude mice and reduced tumor weight (B) (mean ± SEM; *n* = 6; Student’s *t*‐test). (C) The mRNA levels of the GLUT1, HK2, PFK2, PKM2, LDHA, and MCT4 were reduced in SETD1A‐knockdown tumors (mean ± SEM; *n* = 6; Student’s *t*‐test). *P < 0.05, **P < 0.01.

Owing to SETD1A plays a promotional effect on GC cell proliferation by enhancing glycolysis through cooperating with HIF1α in vitro, we further determined whether SETD1A also promoted GC cell tumorigenesis by enhancing metabolism reprogramming *in vivo*. SETD1A‐knockdown and control cells were subcutaneously injected into nude mice, and the xenograft tumor growth rate was monitored, respectively. Downregulated SETD1A in BGC‐823 cells markedly inhibited tumor growth (Fig. 6A) and reduced tumor weight (Fig. 6B). Furthermore, we performed RT‐qPCR to detect the role of SETD1A knockdown on glycolysis *in vivo*. As shown in Fig. 6C, knockdown of SETD1A significantly reduced the mRNA levels of glycolytic genes (GLUT1, HK2, PFK2, PKM2, LDHA, and MCT4) in SETD1A‐knockdown tumors compared to control tumors. These results indicate that SETD1A promotes GC tumorigenesis through enhancing glycolysis *in vivo*.

### 3.8 SETD1A is markedly positively associated with the several glycolytic genes in human GC patients from TCGA dataset

**Fig. 7:**
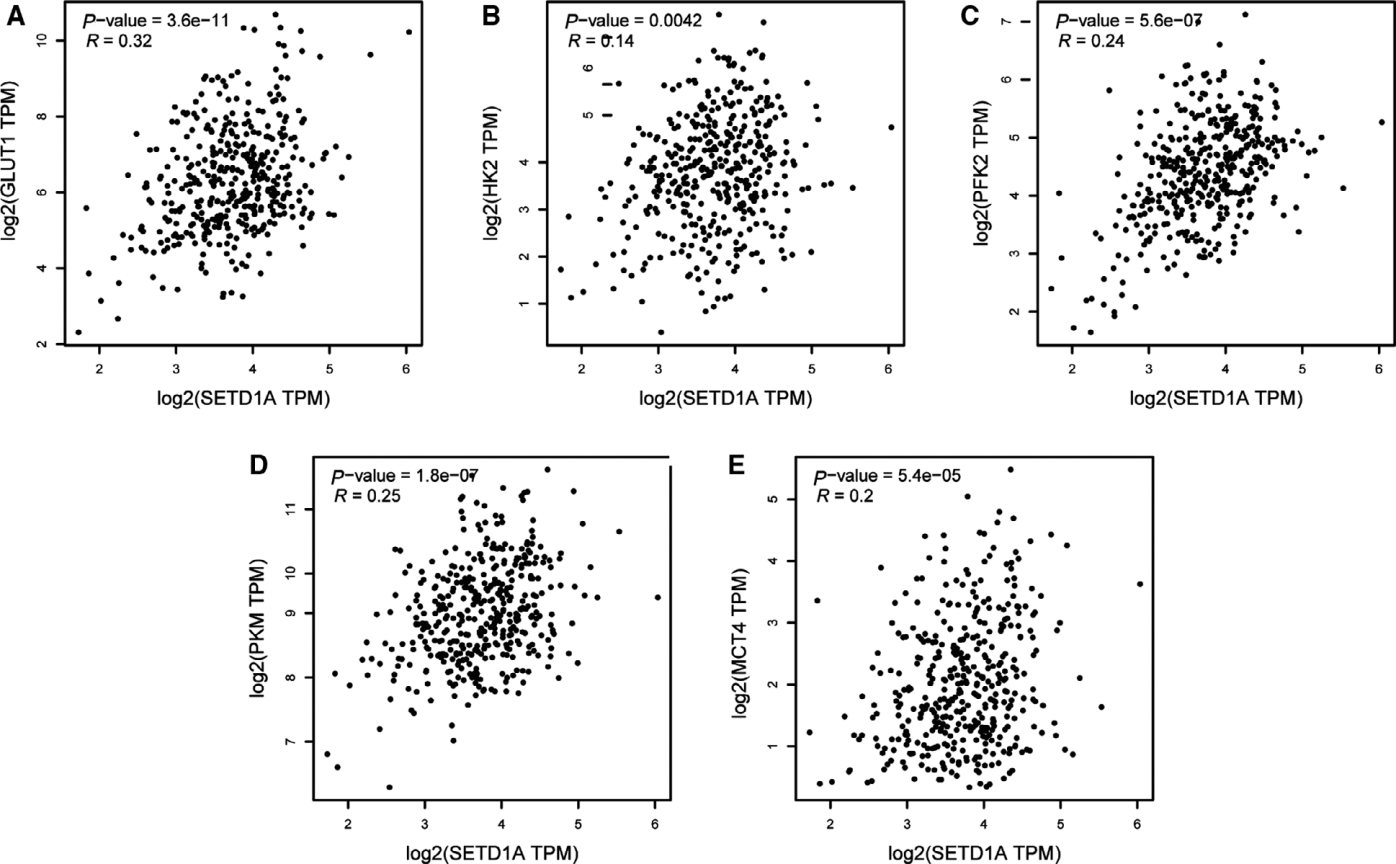
The expression of SETD1A is positively correlated with the expression of glycolytic genes in human gastric cancer specimens from TCGA dataset. Linear regression of SETD1A and glycolytic genes GLUT1 (A), HK2 (B), PFK2 (C), PKM (D), and MCT4 (E) using GC samples from TCGA database obtained by GEPIA.

To determine the relationship between SETD1A and glycolytic genes in human GC specimens, we analyzed the correlation between SETD1A and key glycolytic genes in GC specimens from TCGA dataset. As shown in Fig. 7, SETD1A was markedly positively associated with GLUT1 (Fig. 7A), HK2 (Fig. 7B), PFK2 (Fig. 7C), PKM2 (Fig. 7D), and MCT4 (Fig. 7E). These data strongly indicated our notion that SETD1A promotes GC glycolysis.

## 4 Discussion

Cancer cells frequently favor metabolism reprogramming to fuel cell proliferation and division (Hanahan and Weinberg, 2011; Ward and Thompson, 2012). HIF1α is one of key transcription factors which enhanced glycolytic genes transcription (Semenza, 2010); finding the mediator who regulating HIF1α‐related glycolysis is important for exploiting effective and targeted therapies for cancer. In the current study, we have demonstrated that SETD1A promotes metabolism reprogramming through its interaction with and coactivation of HIF1α to facilitate cancer cell growth and tumorigenesis (Figs 4 and 6). Knockdown of SETD1A decreases aerobic glycolysis, accompanied by reduced expression of glycolytic genes (Fig. 3 and Fig. S1). Furthermore, inhibition of HIF1α‐related glycolysis by downregulating HIF1α slowed down SETD1A‐mediated GC cell proliferation (Fig. 5). Notably, we also discovered there was a markedly positive correlation between SETD1A and several key glycolytic genes in human GC specimens from TCGA dataset (Fig. 7). Altogether, these data provide a potential mechanism that SETD1A works as an oncogene by enhancing glycolysis.

Normal cells uptake glucose, firstly transform to pyruvate via glycolysis in the cytosol and then to carbon dioxide via TCA cycle in the mitochondria under aerobic conditions; however, cancer cell favored glycolysis and dispatched little pyruvate to the oxygen‐consuming mitochondria under anaerobic conditions (Hanahan and Weinberg, 2011). Cancer cells dramatically convert aerobic oxidation to aerobic glycolysis in glucose metabolism (Koppenol *et al.*, 2011). Aerobic glycolysis accelerates the glucose conversion into metabolic intermediates to support anabolic processes in maintaining the high rate of cancer cell proliferation (Schulze and Harris, 2012; Vander Heiden *et al.*, 2009; Vander Heiden *et al.*, 2011). Therefore, inhibition of the intracellular glycolysis could be an effective and targeted approach to reduce cancer cell proliferation. In this report, we demonstrated that knockdown of SETD1A decreased the glycolytic rate in GC cells (Fig. 3). In addition, inhibition of HIF1α suppressed SETD1A overexpression‐induced cell proliferation (Fig. 5), indicating SETD1A‐overexpressing GC cells are more partial to glycolysis. Since systematic HK2 deletion does not exhibit any abnormal physiological consequences (Patra *et al.*, 2013), targeting any key glycolytic enzymes could be a valuable and safe approach for treating solid tumors.

Multiple of evidences showed that HIF1α was a key transcription factor which transcriptionally activates key glycolytic gene expression to enhance glycolysis (Wan *et al.*, 2017). Several transcriptional coactivators which can strengthen HIF1α transactivation activity have been identified (Patra *et al.*, 2013; Perez‐Perri *et al.*, 2016; Wan *et al.*, 2017; Zhao *et al.*, 2014). In the current study, we found that SETD1A could be recruited to and methylate the H3K4 on the glycolytic genes HK2 and PFK2 promoter and acted as a coactivator of HIF1α to strengthen its transactivation activity.

SETD1A, a SET1/MLL H3K4 methyltransferases, can induce gene expression through adding mono‐ to tri‐methyl on H3K4 (Schneider *et al.*, 2005). Previous studies have shown that SETD1A played a key role in embryonic stem cell self‐renewal (Fang *et al.*, 2016) and is required for breast cancer, lung adenocarcinoma, and colorectal cancer development (Fang *et al.*, 2018; Jin *et al.*, 2018; Salz *et al.*, 2015; Salz *et al.*, 2014). In the present study, we provided convincing evidence exhibiting that SETD1A plays critical roles in GC cell proliferation and tumorigenesis by enhancing glycolysis (Figs 2 and 6). The current study also showed that high level of SETD1A is positively associated with poor outcome (Fig. 1), suggesting that SETD1A is positively correlated to gastric cancer. These results indicated that SETD1A may be a potential predictor for GC outcome.

## 5 Conclusions

The current study demonstrates that SETD1A plays a key role in enhancing glycolysis and promoting GC progression by cooperating with HIF1α, suggesting that SETD1A is a valuable molecular target for treatment of GC.

## Conflict of interest

The authors declared no conflict of interest.

## Author contributions

YG and JY contributed to the study concepts, study design, and manuscript edit and review. JW and HC carried out the study design, most experiments, data acquisition and analysis, and manuscript preparation. XX performed the animal and some cellular experiments. All authors read and approved the final manuscript.

## Supporting information


**Fig. S1. **Downregulation of SETD1A decreases glycolysis in AGS cells. (A‐C) Glucose uptake (A), Lactate production (B) and pH values (C) of SETD1A knockdown cells were reduced under normoxic (N) and hypoxic (H) conditions for 24 h compared to control AGS cells (mean ± SEM; n = 3; Student’s t‐test). (D) The protein levels of HK2 and LDHA were remarkedly reduced in SETD1A‐knockdown AGS cells under normoxic (N) and hypoxic (H) conditions for 24 h. (E‐J) The mRNA levels of GLUT1 (E), HK2 (F), PFK2 (G), PKM2 (H), LDHA (I) and MCT4 (J) were remarkedly reduced in SETD1A‐knockdown AGS cells under normoxic (N) and hypoxic (H) conditions for 24 h (mean ± SEM; n = 4; Student’s t‐test). *P < 0.05, ** P < 0.01, *** P < 0.001, **** P < 0.0001.
