# Supplementary figures and images for: Histone methyltransferase SETD1A interacts with HIF1α to enhance glycolysis and promote cancer progression in gastric cancer
Source: Mol Oncol. 2020 Apr 26;14(6):1397–409. doi: 10.1002/1878-0261.12689 (PMC7266269; doi:10.1002/1878-0261.12689)

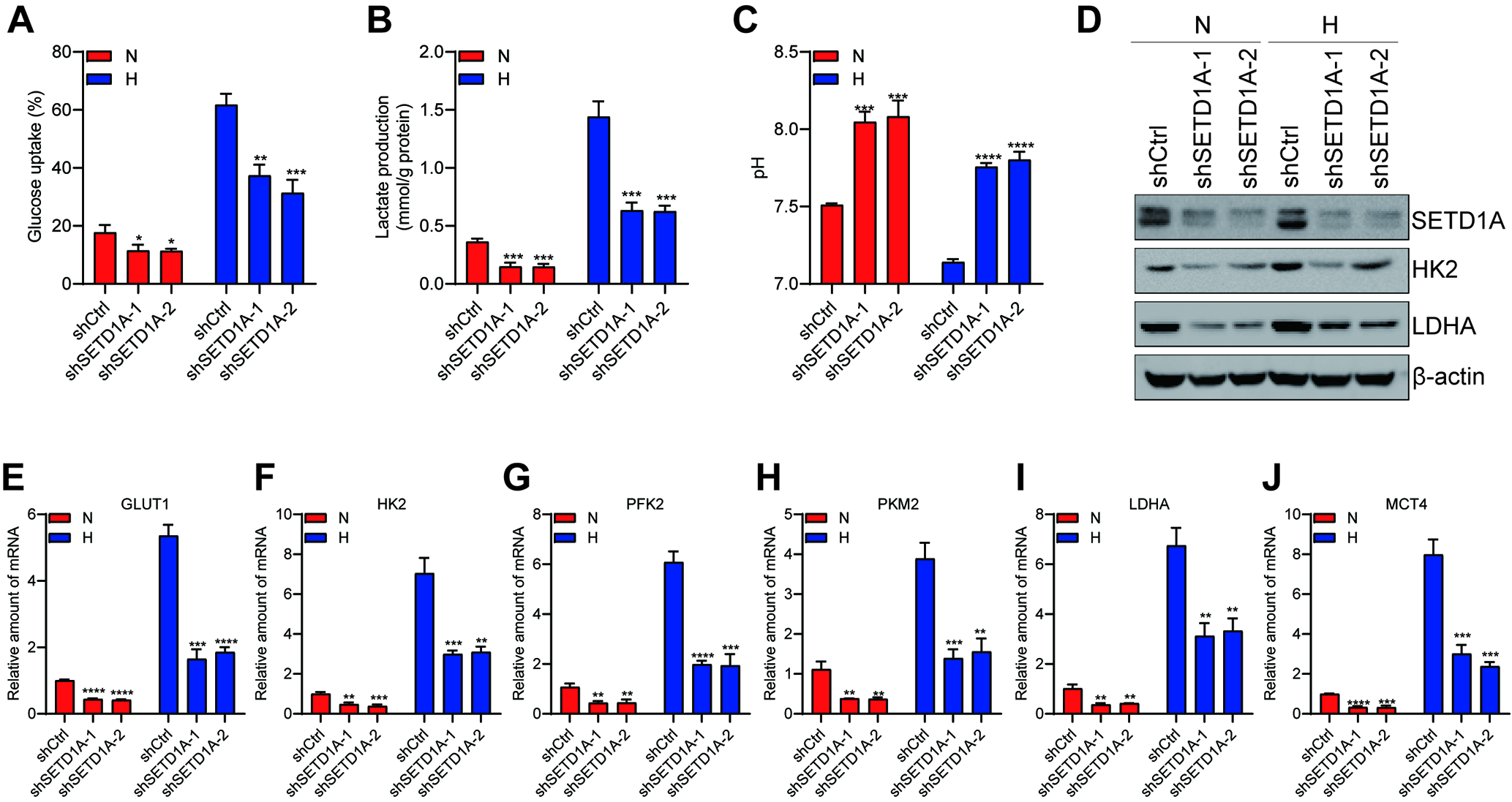

Supplement: Supplementary file 1 — Fig. S1. Downregulation of SETD1A decreases glycolysis in AGS cells. (A‐C) Glucose uptake (A), Lactate production (B) and pH values (C) of SETD1A knockdown cells were reduced under normoxic (N) and hypoxic (H) conditions for 24 h compared to control AGS cells (mean ± SEM; n = 3; Student’s t‐test). (D) The protein levels of HK2 and LDHA were remarkedly reduced in SETD1A‐knockdown AGS cells under normoxic (N) and hypoxic (H) conditions for 24 h. (E‐J) The mRNA levels of GLUT1 (E), HK2 (F), PFK2 (G), PKM2 (H), LDHA (I) and MCT4 (J) were remarkedly reduced in SETD1A‐knockdown AGS cells under normoxic (N) and hypoxic (H) conditions for 24 h (mean ± SEM; n = 4; Student’s t‐test). *P < 0.05, ** P < 0.01, *** P < 0.001, **** P < 0.0001. [file MOL2-14-1397-s001.TIF]
